# Inhibitory Effect of Luteolin on Spike S1 Glycoprotein-Induced Inflammation in THP-1 Cells via the ER Stress-Inducing Calcium/CHOP/MAPK Pathway

**DOI:** 10.3390/ph17101402

**Published:** 2024-10-20

**Authors:** Sonthaya Umsumarng, Sivamoke Dissook, Punnida Arjsri, Kamonwan Srisawad, Pilaiporn Thippraphan, Apiwat Sangphukieo, Patcharawadee Thongkumkoon, Pornngarm Dejkriengkraikul

**Affiliations:** 1 Faculty of Veterinary Medicine, Chiang Mai University, Chiang Mai 50100, Thailand; sonthaya.u@cmu.ac.th; 2 Center for Research and Development of Natural Products for Health, Chiang Mai University, Chiang Mai 50200, Thailand; sivamoke.dis@cmu.ac.th; 3 Department of Biochemistry, Faculty of Medicine, Chiang Mai University, Chiang Mai 50200, Thailand; punnida.dream@gmail.com (P.A.); kamonwan.sri@cmu.ac.th (K.S.); tipprapant@gmail.com (P.T.); 4 Anticarcinogenesis and Apoptosis Research Cluster, Faculty of Medicine, Chiang Mai University, Chiang Mai 50200, Thailand; 5 Center of Multidisciplinary Technology for Advanced Medicine (CMU-TEAM), Faculty of Medicine, Chiang Mai University, Chiang Mai 50200, Thailand; apiwat.sang@cmu.ac.th (A.S.); patcharawadee.t@cmu.ac.th (P.T.)

**Keywords:** bioinformatic analyses, CAMK2A, COVID-19, inflammatory cytokines, transcriptomic analyses

## Abstract

Background/Objectives: The global SARS-CoV-2 outbreak has escalated into a critical public health emergency, with the spike glycoprotein S1 subunit of SARS-CoV-2 (spike-S1) linked to inflammation in lung tissue and immune cells. Luteolin, a flavone with anti-inflammatory properties, shows promise, but research on its effectiveness against long-COVID-related inflammation and spike protein-induced responses remains limited. This study aims to elucidate the underlying mechanisms of inflammation in THP-1 cells induced by the spike-S1. Additionally, it seeks to assess the potential of luteolin in mitigating inflammatory responses induced by the spike-S1 in a THP-1 macrophage model. Methods: The gene expression profiles of spike-S1 in THP-1 cells were analyzed by transcriptome sequencing. The inhibitory effect of luteolin on ER stress and inflammation in spike-S1-induced THP-1 cells was investigated using Western blotting, RT-PCR, and ELISA. Results: The candidate genes (*CAMK2A*, *SIGLEC7*, *PPARGC1B*, *SEC22B*, *USP28*, *IER2*, and *TIRAP*) were upregulated in the spike-S1-induced THP-1 group compared to the control group. Among these, calcium/calmodulin-dependent protein kinase II alpha (CAMK2A) was identified as the most promising molecule in spike-S1-induced THP-1 cells. Our results indicate that the spike S1 significantly increased the expression of ER-stress markers at both gene and protein levels. Luteolin significantly reduced ER stress by decreasing the expression of ER-stress marker genes and ER-stress marker proteins (*p* < 0.01). Additionally, luteolin exhibited anti-inflammatory properties upon spike S1-induction in THP-1 cells by significantly suppressing IL-6, IL-8, and IL-1β cytokine secretion in a dose-dependent manner (*p* < 0.05). Furthermore, our results revealed that luteolin exhibited the downregulation of the MAPK pathway, as evidenced by modulating the phosphorylation of p-ERK1/2, p-JNK and p-p38 proteins (*p* < 0.05). Conclusions: The results from this study elucidate the mechanisms by which the spike S1 induces inflammation in THP-1 cells and supports the use of naturally occurring bioactive compounds, like luteolin, against inflammation-related SARS-CoV-2 infection.

## 1. Introduction

Severe acute respiratory syndrome coronavirus 2 (SARS-CoV-2) infection, also known as COVID-19, has become a serious public health problem worldwide. SARS-CoV-2 targets the human respiratory system and causes severe inflammatory responses [1,2]. The severity of severe acute respiratory syndrome coronavirus 2 (SARS-CoV-2) infection is associated with elevated levels of pro-inflammatory cytokines in the patient’s blood, including IL-1β, IL-6, IL-8, IL-18, TNF-α, MCP-1, and IFN-γ, particularly in patients with severe symptoms [3,4,5]. At present, the pathogenesis of inflammation-related SARS-CoV2 infection has not been fully elucidated but has been found to be potentially associated with the major structural proteins of SARS-CoV-2 (spike protein) [6,7]. Interestingly, during SARS-CoV-2 infection, the binding of the spike protein to the infected cells (mainly lung epithelial cells and immune cells) can activate different pathways rather than a simple entry to the host cell. Spike proteins can also do additional damage by activating toll-like receptors (TLRs), leading to the secretion of pro-inflammatory cytokines independent of viral entry [7,8,9]. Furthermore, activated TLRs involve multiple inflammatory signaling pathways, such as MAPK, JAK-STAT, and NLRP3 inflammasome, that are known to regulate the expression of the genes involved in immunity and inflammation in lung epithelial cells and immune cells [10,11]. Currently, no effective therapy is available for the management of Long-COVID symptoms, and only common drugs for supportive therapies have been used. Therefore, it draws our attention to the search for active compounds from the medicinal herb with potential therapeutic effects to reduce inflammation and relieve Long-COVID symptoms.

Luteolin (3′,4′,5,7-tetrahydroxy flavone) is a flavone naturally occurring as a glycosylated form and is present in different fruits and vegetables [12]. The previous study found that luteolin could inhibit the LPS-induced release of TNF-α, IL-6, and nitric oxide by macrophages [13,14,15]. Moreover, luteolin significantly decreased the LPS-induced secretion of INF-γ, IL-6, COX-2, and iNOS in the alveolar macrophage and peripheral macrophage RAW 264.7 cell lines [16,17]. Therefore, luteolin has a high potential to reduce inflammation from SARS-CoV-2 infection [18,19]. However, there is a limited number of studies regarding the anti-inflammatory properties against inflammation-related long-COVID nor the inhibition of inflammatory responses upon spike protein of SARS-CoV-2 induction. In the present study, we propose to identify key molecular mechanisms underlying the spike protein S1 of SARS-CoV-2-induced THP-1 macrophage cells inflammation and investigate the anti-inflammatory properties of luteolin by determining the inhibition of SP-induced inflammatory gene expressions and their responsible anti-inflammatory pathway using the in vitro THP-1 macrophages cells model. To explore the underlying mechanisms, we induced the THP-1 cells with spike protein S1 of SARS-CoV-2 and compared the relevant gene expression profiles using the transcriptome sequencing technique. In addition, we also compared the relevant gene expression profiles between spike protein S1-induced THP-1 cells and luteolin-treated spike protein S1-induced THP-1 cells using the transcriptome sequencing technique. Bioinformatic analysis revealed that the ER-stress/calcium pathway and inflammatory pathway were more active in spike protein S1-induced THP-1 cells. All of these pathways indicated the presence of calcium/calmodulin-dependent protein kinase II alpha (CAMK2A) and TIR domain-containing adaptor protein (TIRAP). These highly expressed genes were further confirmed by qPCR and Western blot analysis. Furthermore, we investigated the inhibitory effect of luteolin on spike protein S1-induced inflammatory gene expressions and ER-stress/calcium pathway via the inhibition of the MAPK signaling pathway.

The results obtained from this study provide underlying mechanisms of spike protein S1 to induce THP-1 cell inflammation and potentiate the use of naturally occurring bioactive compounds, such as luteolin, against inflammation-related SARS-CoV-2 infection.

## 2. Results

### 2.1. Effect of Luteolin on THP-1 Cell Viability

**Figure 1:**
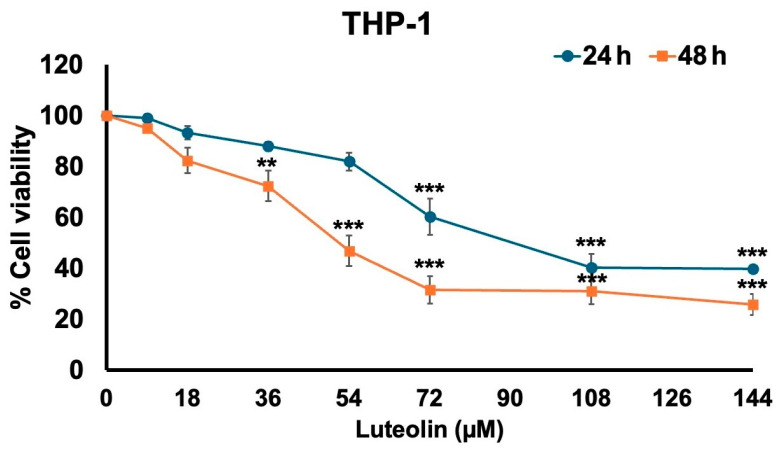
The effect of luteolin on THP-1 cell viability was evaluated. THP-1 cells were treated with luteolin at concentrations of 0, 8, 18, 36, 54, 72, 108, and 144 μM for 24 and 48 h. Cell survival was determined using an MTT assay. The results are presented as means ± S.D. from triplicate samples of three independent experiments. Differences between the means were analyzed using the independent *t*-test. Statistical significance was considered when ** *p* < 0.01 or *** *p* < 0.001 vs. the control group (0 μM of luteolin).

Before evaluating the anti-inflammatory properties of luteolin, the effect of luteolin on the viability of THP-1 cells was assessed. The viability of THP-1 macrophage cells in response to luteolin treatment was measured using the MTT assay. As shown in Figure 1, luteolin decreased the cell viability of THP-1 cells, with 50% inhibitory concentration (IC_50_) values of 98 μM and 49 μM after 24 and 48 h of incubation, respectively. In all experiments, THP-1 cells were treated with luteolin for 24 h before harvest. Luteolin concentrations ranging from 0 to 36 μM were found to be optimal, with cell viability remaining above 80% after 24 h of incubation. Therefore, these concentrations were selected for further experiments. These concentrations were used to investigate the inhibition of spike S1-induced inflammatory gene expressions and the associated anti-inflammatory pathways in an in vitro THP-1 macrophage cell model.

### 2.2. Differential Expression Analysis of Untreated THP-1 Cells and SARS-CoV-2 Spike S1-Induced THP-1

**Figure 2:**
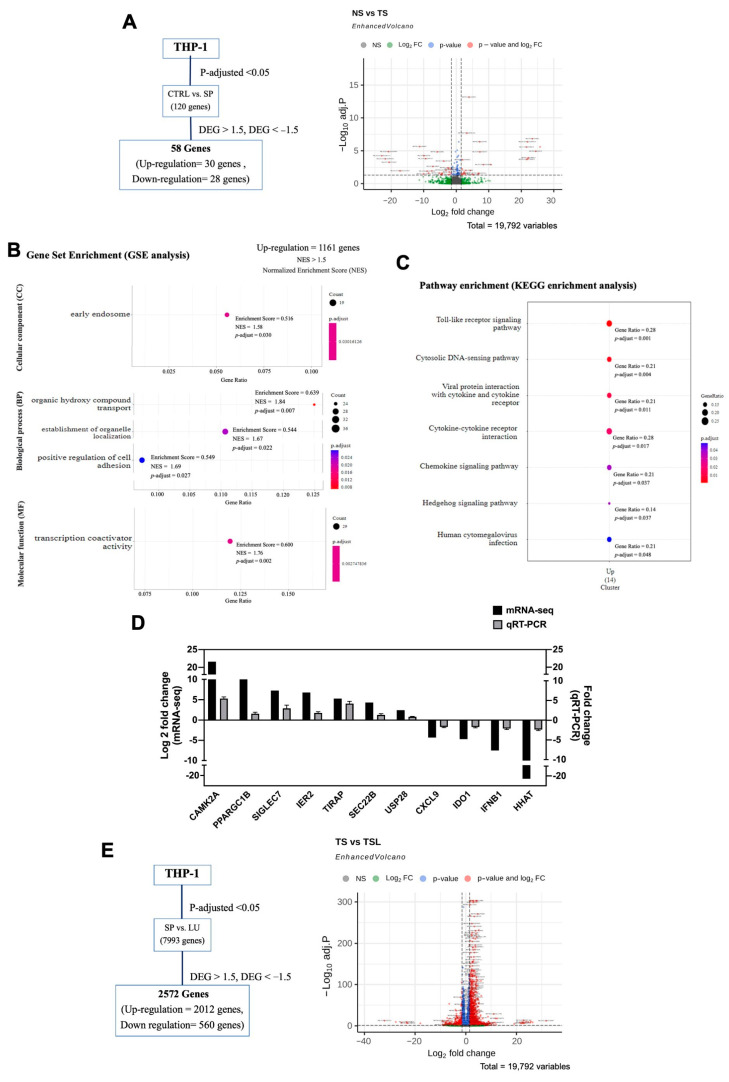
Identification of differentially expressed genes between THP-1 (NS) vs. spike S1-induced THP-1 cells (TS) and spike S1-induced THP-1 cells (TS) vs. spike S1-induced THP-1 treated with luteolin (TSL). (**A**) The volcano diagram of differentially expressed genes between THP-1 and SP1-induced THP-1 cells shows 30 upregulated and 28 downregulated genes. (**B**) David Bioinformatics functional annotation according to gene ontology (GO) in biological process, molecular function, and cellular compartment; (**C**) Pathway enrichment (KEGG enrichment) analysis. (**D**) To validate the differentially expressed genes from mRNA sequencing, the expression levels of seven upregulated and four downregulated genes were examined by using qPCR. (**E**) The volcano diagram of differentially expressed genes between SP1-induced THP-1 (TS) and SP1-induced THP-1 treated with luteolin (TSL) shows 2012 upregulated and 560 downregulated genes.

To investigate the changes in the transcriptome of THP-1 cells, we compared gene expression between untreated THP-1 cells (NS) and SARS-CoV-2 spike S1-induced THP-1 cells (TS) using high throughput mRNA-Seq analysis. As depicted in Figure 2A and Figure S1, the volcano plot analysis identified 58 differentially expressed genes (DEGs) in the SARS-CoV-2 spike S1-induced THP-1 cells (TS) compared to untreated cells (NS). Of these, 30 genes were upregulated, and 28 genes were downregulated (log2-fold change ratio ≥ 1.5 or ≤−1.5 and false discovery rate ≤ 0.05). Gene Ontology (GO) enrichment analysis revealed significant enrichment (*p* < 0.05) in several GO terms, including one for cellular component (CC), three for biological process (BP), and one for molecular function (MF). These terms were mainly associated with early endosome-related genes, organic hydroxy compound transport, establishment of organelle localization, positive regulation of cell adhesion, and transcription coactivator activity genes (Figure 2B). Furthermore, the Kyoto Encyclopedia of Genes and Genomes (KEGG) pathway analysis identified seven significantly enriched pathways (*p* < 0.05), with the most notable being “Viral protein interaction with cytokine and cytokine receptor” and “Cytokine-cytokine receptor interaction” pathways in SARS-CoV-2 spike S1-induce THP-1 cells (Figure 2C). Further examination of DEGs related to viral protein interaction, cytokine receptor, inflammation, and transcription coactivators identified 11 key genes: *CAMK2A*, *SIGLEC7*, *PPARGC1B*, *SEC22B*, *USP28*, *IER2*, and *TIRAP* were upregulated, while *CXCL9*, *IDO1*, *IFNB1*, and *HHAT* were downregulated in SARS-CoV-2 spike S1-induced THP-1 cells.

The accuracy of the expression trends from the mRNA-seq results was validated by qRT-PCR analysis, a conventional method. The mRNA expression of 11 key genes between untreated THP-1 cells (NS) and SARS-CoV-2 spike S1-induced THP-1 cells (TS) was evaluated. The expression trends observed in qRT-PCR confirmed the upregulation of *CAMK2A*, *SIGLEC7*, *PPARGC1B*, *SEC22B*, *USP28*, *IER2*, and *TIRAP*, as well as the downregulation of *CXCL9*, *IDO1*, *IFNB1*, and *HHAT* (Figure 2D), which were consistent with the mRNA-seq results. In this validation, we did not perform statistical analysis due to the differing principles and sensitivity of the two methods.

To assess the reversing effect of luteolin on mRNA expression in SARS-CoV-2 spike S1-induced THP-1 cells, we performed differential gene expression analysis between SARS-CoV-2 spike S1-induced THP-1 cells (TS) and luteolin-treated SARS-CoV-2 spike S1-induced THP-1 cells (TSL). The volcano plot in Figure 2E and Figure S2 revealed a total of 2572 DEGs in luteolin-treated cells, with 2012 genes upregulated and 560 genes downregulated compared to untreated SARS-CoV-2 spike S1-induced cells (log2-fold change ratio ≥ 1.5 or ≤−1.5, false discovery rate ≤ 0.05). Notably, luteolin treatment reversed the expression of the 11 key genes altered by SARS-CoV-2 spike S1 induction (Table 1). Among these genes, CAMK2A is particularly interesting due to its significant differential expression across untreated cells (NS), SARS-CoV-2 spike S1-induced cells (TS), and luteolin-treated cells (TSL). The upregulation of CAMK2A in SARS-CoV-2 spike S1-induced THP-1 cells is associated with endoplasmic reticulum (ER) stress. CAMK2A is known to play a critical role in cellular stress responses, including the activation of ER stress pathways. It has been shown to modulate the unfolded protein response (UPR) and influence the expression of key stress-related proteins such as GRP78/BiP and CHOP, which are important markers of ER stress [20]. This suggests that the upregulation of CAMK2A may contribute to the cellular stress observed in SARS-CoV-2 infections, highlighting its potential role in the pathogenesis of COVID-19.

### 2.3. The Effect of Luteolin on the Expression of ER Stress Markers (CAMK2A/CHOP) in SARS-CoV-2 Spike S1-Induced THP-1 Cells

**Figure 3:**
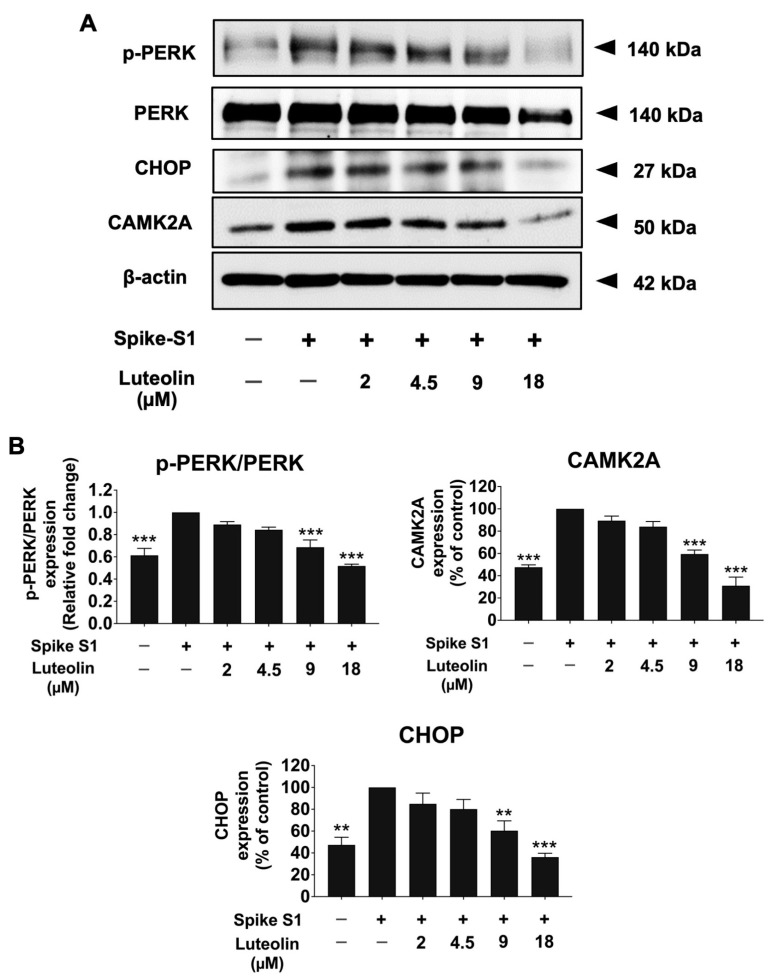
Luteolin inhibited the ER marker (p-PERK, CAMK2A, CHOP) in Spike-S1- induced THP-1 cells. THP-1 cells were pre-treated with luteolin at a concentration of 0–18 μM for 24 h and then exposed to spike S1 for 3 h. The inhibitory effects of luteolin on the ER marker in THP-1 cells were displayed in Western blot (**A**) and band density measurements (**B**). The spike S1-induced THP-1 cells are presented as 100% of control. The results are presented as means ± S.D. from triplicate samples of three independent experiments. Differences between the means were analyzed using one-way ANOVA. Statistical significance was considered when ** *p* < 0.01 or *** *p* < 0.001 vs. the spike S1-induced control group.

To evaluate whether ER stress is triggered upon SARS-CoV-2 spike S1-induced THP-1 cells and whether luteolin can inhibit this stress, we examined the effects of luteolin on ER stress markers, including p-PERK, CAMK2A, and CHOP protein expressions, using the Western blot technique. The results showed that the SARS-CoV-2 spike S1 significantly increased the expression of p-PERK, CAMK2A, and CHOP proteins in SARS-CoV-2 spike S1-induced THP-1 cells when compared with the control group (*p* < 0.001). Notably, luteolin treatment significantly decreased the expression of p-PERK, CAMK2A, and CHOP proteins in a dose-dependent manner in spike S1-exposed THP-1 cells (Figure 3 and Figure S3).

**Figure 4:**
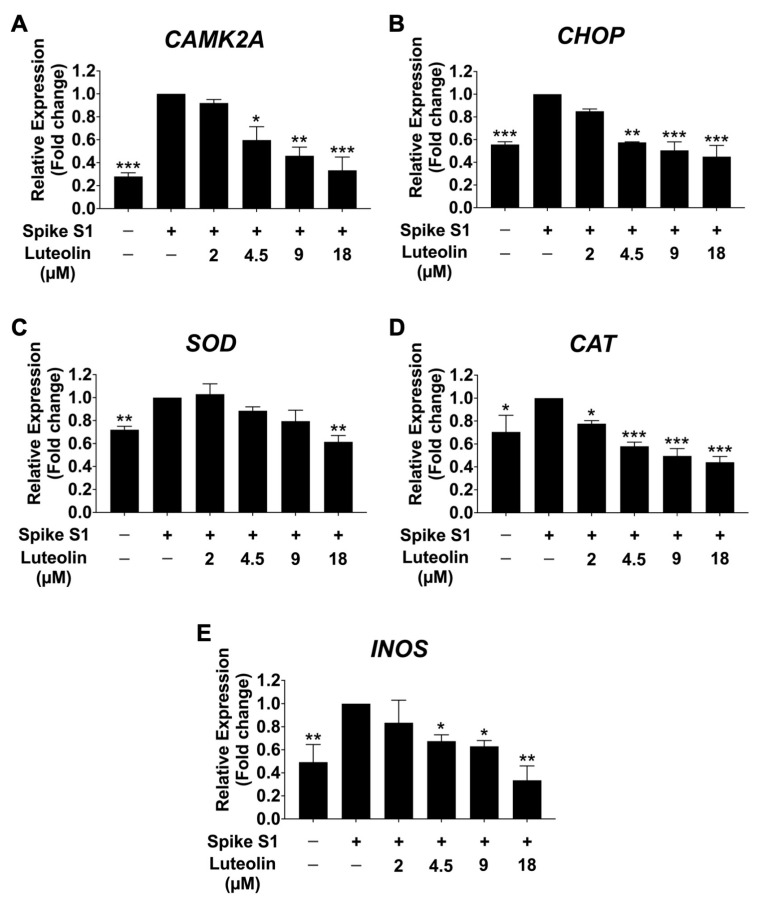
Inhibitory effects of luteolin on the ER marker (*CAMK2A* (**A**), *CHOP* (**B**), *SOD* (**C**), *CAT* (**D**), and *INOS* (**E**)) gene expression in Spike S1-induced THP-1 cells. THP-1 cells were pre-treated with luteolin at a concentration of 0–18 μM for 24 h. Then, the cells were exposed to spike S1 (100 ng/mL) for 3 h. The mRNA expressions were determined using RT-qPCR. The results are presented as means ± S.D. from triplicate samples of three independent experiments. Differences between the means were analyzed using one-way ANOVA. Statistical significance was considered when * *p* < 0.05, ** *p* < 0.01, or *** *p* < 0.001 vs. the spike S1-induced control group.

To further investigate the effect of luteolin on mRNA expression of ER stress-responsive genes (*CAMK2A* and *CHOP*) and oxidative stress marker genes (*SOD*, *CAT*, and *INOS*) in SARS-CoV-2 spike S1-induced THP-1 cells, qRT-PCR was performed. As is shown in Figure 4A,B, the mRNA levels of CAMK2A and CHOP were significantly increased in the spike S1-exposed THP-1 cells compared to the control group (*p* < 0.001). Luteolin significantly reduced ER stress by significantly decreasing the expression of these ER-stress marker genes in a dose-dependent manner. Moreover, the oxidative stress marker genes *SOD*, *CAT*, and *INOS* were significantly upregulated upon SARS-CoV-2 spike S1 induction, as shown in Figure 4C–E. Luteolin treatment significantly reduced the mRNA expression of SOD, CAT, and INOS in a dose-dependent manner in spike S1-induced THP-1 cells. The amplification curves and the cycle threshold point (Ct) values are shown in Figure S4 and Table S1.

### 2.4. The Effect of Luteolin on Inflammatory Cytokine Release in SARS-CoV-2 Spike S1-Induced THP-1 Cells

**Figure 5:**
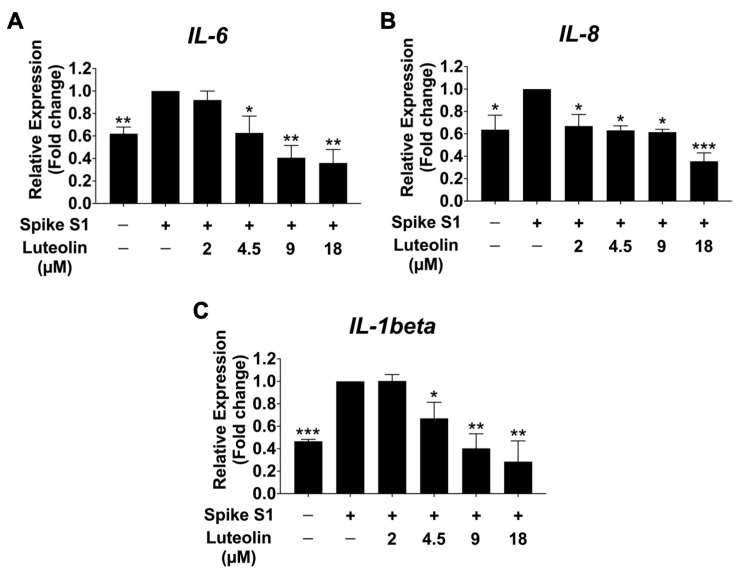
Inhibitory effects of luteolin on the pro-inflammatory cytokines, *IL-6* (**A**), *IL-8* (**B**), *IL-1β* (**C**), gene expression in spike S1-induced THP-1 cells. THP-1 cells were pre-treated with luteolin at a concentration of 0–18 μM for 24 h. Then, the cells were exposed to spike S1 (100 ng/mL) for 3 h. The mRNA expressions were determined using RT-qPCR. The results are presented as means ± S.D. from triplicate samples of three independent experiments. Differences between the means were analyzed using one-way ANOVA. Statistical significance was considered when * *p* < 0.05, ** *p* < 0.01, or *** *p* < 0.001 vs. the spike S1-induced control group.

**Figure 6:**
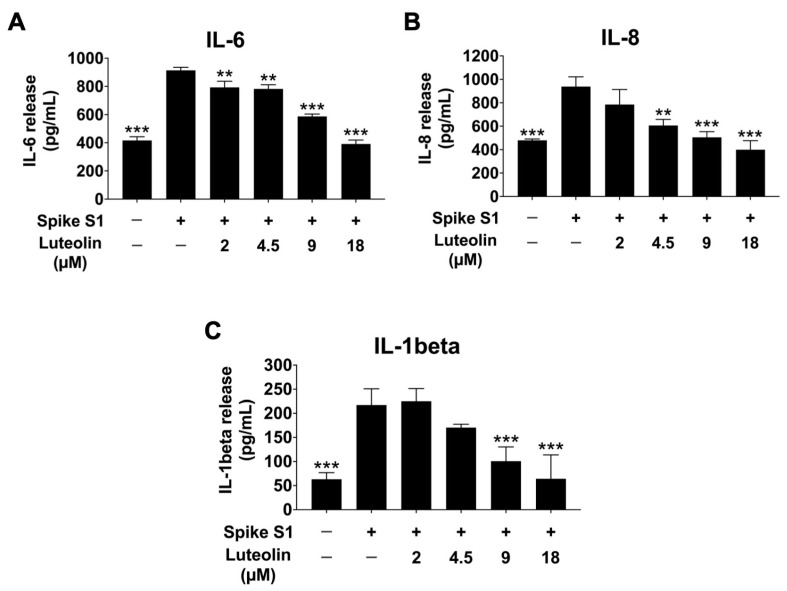
Inhibitory effects of luteolin on the pro-inflammatory cytokine release in Spike S1-induced THP-1 cells. THP-1 cells were pre-treated with luteolin at a concentration of 0–18 μM for 24 h. Then, the cells were exposed to spike S1 (100 ng/mL) for 3 h. The IL-6 (**A**), IL-8 (**B**), and IL-1β (**C**) released into the culture supernatant were examined by ELISA. The results are presented as means ± S.D. from triplicate samples of three independent experiments. Differences between the means were analyzed using one-way ANOVA. Statistical significance was considered when ** *p* < 0.01, or *** *p* < 0.001 vs. the spike S1-induced control group.

All Previous experiments demonstrated that SARS-CoV-2 spike S1 induces ER stress and upregulates the expression of ER stress-responsive genes in THP-1 macrophage cells. This ER stress triggers the release of inflammatory cytokines in macrophages by activating MAPK, NF-κB, and JNK signaling pathways, resulting in the production of pro-inflammatory cytokines such as IL-6, IL-8, and IL-1β, thereby promoting inflammatory responses in macrophages [21,22]. To determine the anti-inflammatory effects of luteolin on SARS-CoV-2 spike S1-induced THP-1 cells inflammation, we examined the mRNA levels of IL-6, IL-8, and IL-1β, as well as the release of these pro-inflammatory cytokines into the culture supernatant, using qRT-PCR and ELISA. As is shown in Figure 5 and Figure 6, the SARS-CoV-2 spike S1 protein significantly increased the expression of IL-6, IL-8, and IL-1β at both mRNA and protein levels. Luteolin treatment significantly reduced the expression of these pro-inflammatory cytokines in a dose-dependent manner, as demonstrated by a decrease in the mRNA levels of IL-6, IL-8, and IL-1β genes in Spike S1-induced THP-1 cells (Figure 5). The amplification curves and the cycle threshold point (Ct) values are shown in Figure S5 and Table S2. Additionally, the release of cytokines IL-6, IL-8, and IL-1β was significantly decreased in luteolin-treated spike S1-induced THP-1 cells compared to the control group (*p* < 0.001), as shown in Figure 6. These results show the concentrations of IL-6, IL-8, and IL-1β in quantitative terms (pg/mL) and indicate that luteolin exhibits significant anti-inflammatory properties by reducing the expression and release of pro-inflammatory cytokines IL-6, IL-8, and IL-1β in SARS-CoV-2 spike S1-induced THP-1 cells, suggesting its potential as a therapeutic agent against inflammation induced by SARS-CoV-2 spike S1 protein.

### 2.5. The Effect of Luteolin on MAPK Signaling Pathway in SARS-CoV-2 Spike S1-Induced THP-1 Cells

**Figure 7:**
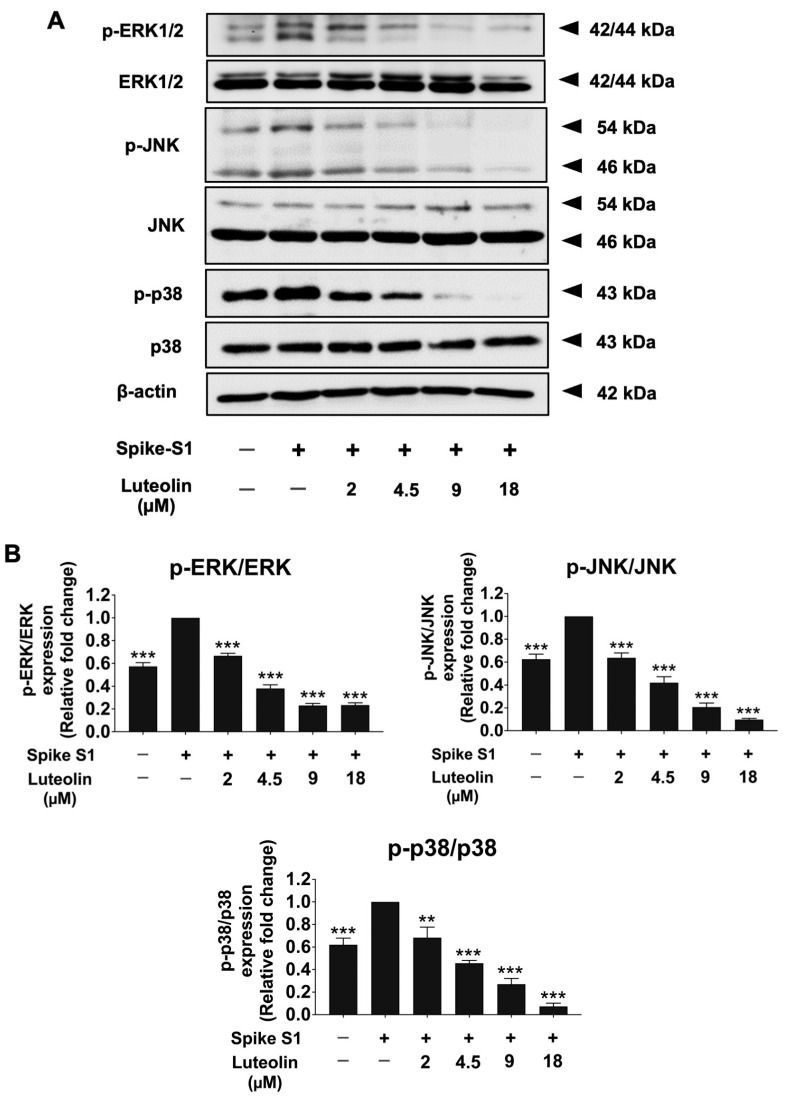
Luteolin inactivated the ERK/JNK/p38 signaling pathway in spike-S1- induced THP-1 cells. THP-1 cells were pre-treated with luteolin at a concentration of 0–18 μM for 24 h and then exposed to spike S1 for 3 h. The inhibitory effects of luteolin on the phosphorylation of ERK, JNK, and p38 proteins in THP-1 cells were displayed in Western blot (**A**) and band density measurements (**B**). The spike S1-induced THP-1 cells are presented as 100% of control. The results are presented as means ± S.D. from triplicate samples of three independent experiments. Differences between the means were analyzed using one-way ANOVA. Statistical significance was considered when ** *p* < 0.01 or *** *p* < 0.001 vs. the spike S1-induced control group.

To explore the upstream regulatory pathways underlying the anti-inflammatory effects of luteolin in SARS-CoV-2 spike S1-induced THP-1 cells, we investigated the protein expression levels of key components in the MAPK signaling pathway using the Western blot analysis. As shown in Figure 7, spike S1 significantly increased the phosphorylation of ERK, JNK, and p38 proteins in THP-1 cells compared to the control group (*p* < 0.001). Luteolin treatment markedly inhibited the phosphorylation of these proteins in spike S1-exposed THP-1 cells in a dose-dependent manner. These findings suggest that luteolin exerts its protective effects against spike S1-induced inflammation by inhibiting the MAPK signaling pathway, specifically through the suppression of ERK, JNK, and p38 protein phosphorylation (Figure 7A,B and Figure S6). In conclusion, luteolin’s anti-inflammatory properties are mediated by the downregulation of MAPK signaling, highlighting its potential as a therapeutic agent in mitigating spike S1-induced inflammation in THP-1 cells.

## 3. Discussion

The high production of pro-inflammatory cytokines in SARS-CoV-2-infected patients is a major contributor to the disease’s severity, particularly in severe cases where acute respiratory distress syndrome (ARDS) can progress to pulmonary fibrosis [5,23]. Given the emergence of viral variants and the serious post-infection complications, there is an urgent need for therapeutic agents that can broadly target the molecular mechanisms involved in SARS-CoV-2 pathology. Macrophages, the most abundant immune cells in lung tissue, play a crucial role in the inflammatory response during SARS-CoV-2 infection by releasing cytokines (e.g., IL-6, IL-1β, TNF-α, IL-12), chemokines (e.g., CXCL8), growth factors (e.g., VEGF, angiopoietins), and enzymes [7,24]. Previous studies have shown that the SARS-CoV-2 spike glycoprotein can activate human lung macrophages, leading to increased expression of CXCL8, IL-6, TNF-α, and IL-1β, which contributes to lung inflammation [6,25]. Thus, inhibiting macrophage-driven inflammation is a promising strategy to mitigate the severe consequences of SARS-CoV-2 infection.

Flavonoids, particularly luteolin, have garnered attention due to their wide range of pharmacological properties, including anti-inflammatory, antioxidant, and anti-cancer effects [26]. Luteolin, abundant in various medicinal plants and herbs, has been shown to exert strong antioxidant activity by scavenging free radicals and inducing the expression of endogenous antioxidant enzymes (e.g., SOD, CAT, GPx, GSH) [27]. It also suppresses inflammation in LPS-stimulated macrophages by inhibiting the NF-κB and AP-1 pathways [16]. In our study, luteolin concentrations of 0–35 μM were found to be non-cytotoxic to THP-1 cells after 24 h of incubation. These concentrations were thus selected for further investigation into the anti-inflammatory effects of luteolin in SARS-CoV2 spike S1-induced THP-1 macrophages.

We aimed to identify the key molecular mechanisms underlying SARS-CoV-2 spike S1-induced inflammation in THP-1 macrophages and to elucidate the anti-inflammatory properties of luteolin. Using mRNA sequencing, we compared the gene expression profiles between untreated cells (NS), spike S1-induced cells (TS), and luteolin-treated spike S1-induced cells (TSL). The bioinformatics analysis between NS and TS revealed 58 differentially expressed genes (DEGs) (*p* < 0.05) in the TS group compared to the NS group. GO enrichment analysis identified five gene groups associated with specific GO terms, and KEGG pathway analysis revealed that seven pathways were significantly enriched in THP-1 macrophage cells following treatment with spike S1 glycoprotein. Based on these findings, we selected the most relevant genes and focused on those involved in cytokine production and the inflammatory process. To investigate the genes altered by SARS-CoV-2 spike S1 induction and assess the reversing effect of luteolin, mRNA-seq analysis was performed on both TS and TSL. The bioinformatics analysis between TS and TSL revealed 2012 DEGs (*p* < 0.05) in TSL compared to TS. Among these, 11 key genes that showed significant reversals in expression following luteolin treatment (Table 1) were selected for further investigation. Of particular interest is CAMK2A, which was significantly upregulated in TS compared to NS and significantly downregulated in TSL compared to TS.

CAMK2A, identified as one of the most upregulated genes in spike S1-induced THP-1 cells, is known to induce ER stress and promote inflammatory pathways. The upregulation of CAMK2A in response to spike S1 protein may be driven by pathogen-associated molecular patterns (PAMPs), which trigger ER stress and subsequent immune responses, leading to the production of pro-inflammatory cytokines. Previous studies have identified CAMK2A as a pathological mediator of ER stress, oxidative stress, and mitochondrial dysfunction, with CAMK2A inhibition emerging as a promising therapeutic avenue for treating certain diseases, such as cystic diseases [20]. The lipopolysaccharide (LPS) and mimic of double-stranded viral RNA (poly I:C) have been reported to induce inflammation through the ER stress-induced calcium-CHOP pathway in RAW 264.7 macrophage cells [28,29]. Our findings indicate that spike S1 protein induces ER stress in THP-1 cells by activating the phosphorylation of PERK and increasing the expression of CAMK2A and CHOP, which are markers of ER stress. Additionally, spike S1 protein was found to enhance inflammation by upregulating the gene expression and secretion of IL-6, IL-8, and IL-1β. Furthermore, the elevated CAMK2A levels activated the MAPK signaling pathway, as evidenced by increased phosphorylation of Erk1/2, JNK, and p38 proteins.

Luteolin has previously been reported to inhibit ER stress-induced neuroinflammation in Alzheimer’s disease mouse model [30] and to suppress lipotoxicity-induced NLRP3 inflammasome activation in macrophages by reducing ER stress [31]. Its anti-inflammatory properties have been demonstrated in LPS-stimulated macrophages, where it inhibited the release of TNF-α, IL-6, and nitric oxide [32]. Moreover, luteolin significantly decreased the secretion of INF-γ, IL-6, COX-2, and iNOS in both alveolar and peripheral macrophage RAW 264.7 cell lines [17]. These properties suggest that luteolin could effectively reduce inflammation associated with SARS-CoV-2 infection by downregulating pro-inflammatory cytokines (IL-1β, IL-6, IL-8, IL-18, TNF-α, MCP-1, and IFN-γ) [33,34]. Luteolin inhibited MAPK and NF-κB pathways and attenuated the pulmonary inflammatory response in mice acute lung injury models [35,36]. Moreover, luteolin, an active compound in anti-inflammatory traditional medicines, is among the most commonly prescribed herbs for the treatment or prevention of COVID-19 [37,38]. However, there is a limited number of studies regarding the anti-inflammatory properties against inflammation-related long-COVID nor the inhibition of inflammatory responses upon spike protein of SARS-CoV-2 induction.

Our study further highlights the anti-inflammatory properties of luteolin in the context of SARS-CoV-2 spike S1-induced inflammation. Luteolin significantly reduced spike S1-induced ER stress by inhibiting the phosphorylation of PERK, leading to decreased mRNA expression of CAMK2A, CHOP, SOD, CAT, and iNOS. This reduction in ER stress markers was accompanied by a decrease in CAMK2A and CHOP protein levels in spike S1-induced THP-1 cells. Given that the MAPK signaling acts as both an effector and modulator of the ER stress response, triggering cellular inflammation, luteolin’s inhibition of this pathway is particularly noteworthy. Our findings show that luteolin effectively attenuated the spike S1-induced inflammatory response by downregulating the MAPK pathway, as evidenced by the reduced phosphorylation of Erk1/2, JNK, and p38 proteins. Consequently, luteolin treatment led to a significant decrease in the expression and secretion of inflammatory cytokines, such as IL-6, IL-8, and IL-1β, both the mRNA and protein levels.

The implications of these findings for COVID-19 and long-COVID are profound. The ability of luteolin to inhibit both ER stress and the MAPK signaling pathway, which are critical in the pathogenesis of many inflammatory diseases, suggests that luteolin could serve as a therapeutic option to mitigate inflammation-related complications associated with SARS-CoV-2 infection. Given the role of excessive inflammation in the progression of severe COVID-19, luteolin’s anti-inflammatory properties could help reduce the severity of acute symptoms and potentially prevent the progression to ARDS. Moreover, long-COVID, characterized by persistent symptoms and chronic inflammation, may also benefit from luteolin treatment. The persistent activation of inflammatory pathways in long-COVID patients, particularly through mechanisms involving ER stress and MAPK signaling, suggests that luteolin could help alleviate ongoing inflammation and improve recovery outcomes. Given its broad range of anti-inflammatory effects, luteolin could be integrated into treatment regimens aimed at both acute COVID-19 management and long-term recovery. The findings from this study warrants further investigation into the clinical applications of luteolin for treating COVID-19 and its long-term complications.

While our study provides significant insights into the anti-inflammatory effects of luteolin in SARS-CoC-2 spike S1-induced THP-1 cells, there are some limitations to consider. First, the in vitro nature of this study means that the results may not fully translate to in vivo systems. Therefore, future studies should include animal models and clinical trials to validate the therapeutic potential of luteolin in COVID-19 and long COVID patients. Second, while we identified key signaling pathways and genes affected by luteolin, further research is needed to explore the broader transcriptomic and proteomic changes induced by luteolin, particularly in different cell types and tissues involved in COVID-19 pathology. Lastly, the potential interactions of luteolin with other therapeutic agents used in COVID-19 treatment should be explored to ensure that combination therapies are both safe and effective. Future research should also investigate the optimal dosing, administration routes, and timing of luteolin treatment to maximize its therapeutic benefits.

In conclusion, the combination of current COVID-19 medications with luteolin may have a synergistic effect in enhancing anti-inflammatory responses and promoting innate immune activity. This study elucidates the mechanisms by which the spike protein S1 induces inflammation in THP-1 cells and supports the potential use of naturally occurring bioactive compounds, such as luteolin, in combating inflammation-related SARS-CoV-2 infection. Furthermore, these findings highlight the innovative potential of luteolin for future anti-inflammatory drug development.

## 4. Materials and Methods

### 4.1. Reagents and Chemicals

Roswell Park Memorial Institute (RPMI)–1640 medium, fetal bovine serum (FBS), 2.5% trypsin, and penicillin–streptomycin were obtained from Gibco BRL Company (Grand Island, NY, USA). The protease inhibitor cocktail, RIPA lysis buffer, the reagent of Coomassie PlusTM Protein Assay, and the enhanced chemiluminescence (ECL) reagent were purchased from Thermo Fisher Scientific (Rockford, IL, USA). MTT reagent and primary antibody for anti-β-actin were purchased from Sigma-Aldrich (St. Louis, MO, USA). The primary antibodies for Western blot analysis against p-PERK (cat. no. 3179S), PERK (cat. no. 3192S), CHOP (cat. no. 2895S), CamK2A (cat. no. 3357S), p-ERK1/2 (cat. no. 4377S), ERK1/2 (cat. no. 4696S), p-JNK (cat. no. 9255S), JNK (cat. no. 9252S), p-p38 (cat. no. 4631S), and p38 (cat. no. 9212S) were obtained from Cell Signaling Technology (Beverly, MA, USA). The recombinant human coronavirus SARS-CoV-2 spike glycoprotein S1 (Active) or Spike S1 (cat. no. ab273068) was purchased from Abcam (Cambridge, UK). The Spike S1 glycoprotein was expressed in CHO cells, had >90% purity, and demonstrated biological activity.

### 4.2. Cell Cultures

THP-1 cells (TIB-202TM) were purchased from the American Type Culture Collection (ATCC, Manassas, VA, USA). The cells were cultured in RPMI 1640 medium supplemented with 10% FBS, 2 mM L-glutamine, 50 U/mL penicillin, and 50 μg/mL streptomycin at 37 °C in a humidified 5% CO_2_ atmosphere. THP-1 macrophage differentiation was induced by exposing the cells to 10 ng/mL of the phorbol 12-myristate 13-acetate (PMA) in DMSO for 24 h, followed by the previously described protocol [39]. Briefly, for the resting condition, cells were kept in the presence of PMA for 24 h in a normal growth medium, then changed to PMA-free RPMI during a resting stage for another 24 h. PMA-treated macrophages were maintained in a 5% CO_2_ humidified incubator at 37 °C. THP-1 was cultured between 10–20 passages and applied to use in all subsequent experiments.

### 4.3. Cell Viability Assay

The effect of the luteolin against PMA-treated THP-1 macrophages was determined using a 3-(4,5-dimethylthiazol-2-yl)-2,5-diphenyltetrazolium bromide (MTT) assay. THP-1 cells were seeded at a density of 6.5 × 10^3^ cells/well in a culture medium, and the cells were then treated with increasing concentrations of luteolin (0, 8, 18, 36, 54, 72, 108, and 144 μM) for 24 and 48 h. Following the luteolin treatment, the cells were exposed to 10 μL of 0.5 mg/mL MTT solution in phosphate-buffered saline (PBS) and incubated for 4 h. The culture medium was then removed, and the cells were resuspended with 200 μL of DMSO to dissolve the MTT formazan crystals. Absorbance was then measured at 540 nm for the measurement wavelength and 630 nm for the reference wavelength using a UV-visible spectrophotometer [40,41]. The assay was conducted in triplicate at each concentration of luteolin. Cell viability was determined by comparing the absorbance values to those of the control group, and the percentage was expressed as a percentage relative to the control.

### 4.4. Total RNA Isolation and Expression Analysis

THP-1 cells were treated with, or without SARS-CoV-2 spike S1 protein for 3 h, and THP-1 cells were pretreated with luteolin at a concentration of 36 μM (the maximum safe dose that maintains THP-1 cell viability at greater than 80%) for 24 h and then exposed to 100 ng/mL of Spike S1 for 3 h. RNA was extracted using TRIzol reagent (GIBCO-BRL, Life Technologies, Grand Island, NY, USA) according to the manufacturer’s instructions. mRNA-seq libraries were constructed, and the transcriptome was sequenced on an Illumina sequencing platform (Macrogen Inc., HiSeq 2500, Seoul, Republic of Korea). All the raw data have been deposited under the Gene Expression Omnibus (GEO).

### 4.5. mRNA Sequencing Data Analysis

Raw mRNA sequencing data obtained from MACROGEN Co., Ltd., Republic of Korea, were processed through an extensive bioinformatics pipeline. Initially, sequencing reads were trimmed using Cutadapt (version 3.4) to eliminate adapters and low-quality bases. The cleaned reads were then aligned to the human reference genome (hg38) using HISAT2 (version 2.2.1) to ensure accurate mapping. Following alignment, SAMtools (version 1.12) was utilized to sort and convert SAM files into BAM format. Gene expression quantification and transcript assembly were performed using StringTie (version 2.1.4), generating gene transfer format (GTF) files for each sample. These GTF files were subsequently merged into a unified transcriptome assembly and compared to the reference annotation using GffCompare (version 0.11.6). Differential expression analysis was conducted within the R environment using DESeq2 (version 1.34.0). Data visualization, including the generation of volcano plots, was achieved using the EnhancedVolcano R package (version 1.10.0). Furthermore, Gene Set Enrichment (GSE) and KEGG pathway enrichment analyses were performed using the clusterProfiler package (version 4.0.5) and visualized with the enrichplot package (version 1.12.2). The results of the enrichment analyses were further illustrated using the dotplot function from the ggplot2 package (version 3.3.5).

### 4.6. Determination of Gene Expressions by RT-qPCR Analysis

To determine the effect of luteolin on the mRNA level, THP-1 cells were pretreated with luteolin (0–18 μM) for 24 h and then exposed to 100 ng/mL of Spike S1 for 3 h. Then, the total mRNA was isolated using a Qiazol reagent. The concentration and purity of total RNA were detected using Spectrophotometers (Thermo Fisher Scientific, NanoDrop™ 2000/2000c, Waltham, MA, USA). The cDNA was obtained via reverse transcription (Eppendorf, Mastercycler^®^ nexus gradient, Hamburg, Germany). Quantitative real-time PCR technique was determined using a qRT-PCR (Thermo Fisher Scientific, ABITM 7500 Fast & 7500 Real-Time PCR, Waltham, MA, USA). Gene expressions were analyzed using a real-time PCR machine (Applied Biosystems, QuantStudio 6 Flex real-time PCR machine system software system software v.1.0, Waltham, MA, USA). The 2^−ΔΔCT^ method with normalization to GAPDH and controls was used for the calculation of results.

The primer sequences used for amplifying Calcium/Calmodulin Dependent Protein Kinase II Alpha (CAMK2A), PPARG Coactivator 1 Beta (PPARGC1B), Sialic Acid Binding Ig Like Lectin 7 (SIGLEC7), Immediate Early Response 2 (IER2), Toll-Like Receptor Adaptor Protein (TIRAP), SEC22 Homolog B, Vesicle Trafficking Protein (SEC22B), Ubiquitin Specific Peptidase 28 (USP28), Hedgehog Acyltransferase (HHAT), Interferon Beta 1 (IFNB1), Indoleamine 2,3-Dioxygenase 1 (IDO1), C-X-C Motif Chemokine Ligand 9 (CXCL9), C/EBP-homologous protein (CHOP), Superoxide dismutase (SOD), Catalase (CAT), and glyceraldehyde-3-phosphate dehydrogenase (GAPDH) primer sequences were supplied from Humanizing Genomics Macrogen, Geumcheongu, Seoul, Republic of Korea. All primer sequences used in this study are shown in Table 2.

### 4.7. Determination of Cytokine Release

Firstly, THP-1 cells (6.5 × 10^3^ cells/well) were seeded in a 6-well plate and incubated overnight. The cells were then pre-treated with various concentrations of luteolin (0–18 μM) for 24 h, followed by stimulation with Spike S1 (100 ng/mL) for a further 3 h. The cultured medium was collected for ELISA testing. The cytokine releases, IL-6, IL-8, and IL-1β, in the cultured medium were examined using an ELISA kit (Biolegend, San Diego, CA, USA). The detection protocol was followed according to the manufacturer’s instructions, and the absorbance was measured at 450 and 570 nm. The cytokine release into the culture medium was calculated and compared for each standard curve.

### 4.8. Western Blot Analysis

The THP-1 cells were lysed using RIPA lysis buffer (100 μL/well) and centrifuged at 10,000 rpm for 10 min at 4 °C. The Bradford assay was used to measure the concentration of the protein samples. Equal quantities of protein samples (20 μg) were loaded on a 12% SDS-gel, resolved using SDS-PAGE, and transferred to 0.45 μm PVDF/nitrocellulose membranes, which were soaked in methanol for 10 min in advance. Next, the membranes were blocked with 5% BSA in 0.05% TBS-Tween (TBS-T) for 1 h at room temperature, washed 3 times with 0.05% TBS-T, and incubated with primary antibodies against p-PERK (cat. no. 3179S), PERK (cat. no. 3192S), CHOP (cat. no. 2895S), CamK2A (cat. no. 3357S), p-ERK1/2 (cat. no. 4377S), ERK1/2 (cat. no. 4696S), p-JNK (cat. no. 9255S), JNK (cat. no. 9252S), p-p38 (cat. no. 4631S), p38 (cat. no. 9212S), and β-actin (cat. no. A2228) overnight at 4 °C. Next, after washing the membranes 4 times with 0.5% TBS-T, the membranes were incubated with goat anti-mouse (cat. no. 7076S) or rabbit HRP-conjugated (cat. no. 7074S) secondary antibody for 2 h at room temperature and were then washed 4 times with 0.05% TBS-T. The protein bands were visualized using a chemiluminescence imaging system following treatment with HRP substrate (ECL) for 1–4 min and then exposed to iBright™ CL-1500 imaging system (Thermo Fisher Scientific, Waltham, MA, USA). β-actin was normalized and used as the loading control. Band density levels were analyzed using Image J 1.410. The band density of target proteins was normalized to the band density of β-actin before calculating the percentage expression of each protein, with the spike S1 induction group set to 100% as a reference. The relative fold change of the phosphorylated and total forms of the target proteins was calculated by first normalizing the band density of both the phosphorylated and total forms. Then, the normalized band density of the phosphorylated form was divided by the total form, with the spike S1 induction group set to 1 as a reference for calculating the relative fold change.

### 4.9. Statistical Analysis

Statistical analysis was performed using Prism version 8.0 software based on three independent experiments. All experiments were conducted in triplicate independent trials to ensure reproducibility. The independent *t*-test and one-way ANOVA with Dunnett’s test were employed for data analysis across various experiments, including cell viability assay, ELISA, and qRT-PCR, as well as Western blot assay data. The data were reported as mean ± standard deviation (mean ± S.D.). Statistical significance was established at * *p* < 0.05, ** *p* < 0.01, and *** *p* < 0.001.

## Figures and Tables

**Table 1:** The reverse effect of luteolin on 11 key genes mRNA expression in SARS-CoV-2 spike S1-induced THP-1 cells.

Gene Name	Log2 Fold Change		*p*-adj	
	NS vs. TS Gene	TS vs. TSL Gene	NS vs. TS Gene	TS vs. TSL Gene
NM_171825|*CAMK2A*	21.61	−23.36	2.7 × 10^−6^	4.9 × 10^−10^
NM_001172698|*PPARGC1B*	10.69	−8.11	1.3 × 10^−3^	1.3 × 10^−3^
NM_016543|*SIGLEC7*	7.25	−7.76	1.5 × 10^−5^	8.4 × 10^−9^
MSTRG.2975|*USP28*	6.76	−5.39	3.1 × 10^−2^	8.2 × 10^−3^
MSTRG.7674|*IER2*	5.29	−5.82	4.5 × 10^−2^	3.2 × 10^−4^
MSTRG.3069|*TIRAP*	4.30	−3.34	1.0 × 10^−2^	4.6 × 10^−3^
MSTRG.904|*SEC22B*	2.44	−1.77	1.6 × 10^−2^	1.2 × 10^−2^
NM_001170587|*HHAT*	−20.65	31.70	0.01	7.4 × 10^−13^
MSTRG.15555|*IFNB1*	−7.50	3.06	0.01	1.4 × 10^−1^
MSTRG.15072|*IDO1*	−4.32	5.66	0.03	5.7 × 10^−6^
MSTRG.11698|*CXCL9*	−4.72	7.08	0.04	5.6 × 10^−7^

**Table 2:** Primer sequences were used in this study to determine gene expressions using RT-qPCR analysis.

Gene Product	Primer Sequences	References
*CAMK2A*	Forward: 5′-ACC AGC TCT TCG AGG AAT TG-3′ Reverse: 5′-GTG ACC AGG TCG AAG ATC AG-3′	[42]
*PPARGC1B*	Forward: 5′-ATG ACT CCG AGC TCT TCC AG-3′ Reverse: 5′-CGA AGC TGA GGT GCA TGA TA-3′	[43]
*SIGLEC7*	Forward: 5′-AAG AAG CCA CCA ACA ATG AG-3′ Reverse: 5′-CAG TTA GAC AAG AGG AAT AAG TTC-3′	[44]
*IER2*	Forward: 5′-CCA AAG TCA GCC GCA AAC GA-3′ Reverse: 5′-TTT CTT CCA GAC GGG CTT TCT TGC-3′	[45]
*TIRAP*	Forward: 5′-CTC TGA GAA TAA GAT GTT TCC-3′ Reverse: 5′-ACG CAG ACG TCA TAG TCT TT-3′	[46]
*SEC22B*	Forward: 5′-GGC CAA TAG ACG AGA TCT GT-3′ Reverse: 5′-CTT AGT CAA CCT GTG CCA GC-3′	[47]
*USP28*	Forward: 5′-CCG AAC AGT TCT GCG TGC T-3′ Reverse: 5′-CAC CGG CTG TGA AGC TGA-3′	[48]
*HHAT*	Forward: 5′-GGG TGC TTG TTT CTG AGA TTT G-3′ Reverse: 5′-GGG TAC ACT ATC CTG TGG TTT C-3′	[49]
*IFNB1*	Forward: 5′-CAG CAA TTT TCA GTG TCA GCA AGC T-3′ Reverse: 5′-TCA TCC TGT CCT TGA GGC AGT AT-3′	[50]
*IDO1*	Forward: 5′-TCA CAG ACC ACA AGT CAC AG-3′ Reverse: 5′-GCA AGA CCT TAC GGA CAT CT-3′	[51]
*CXCL9*	Forward: 5′-CCA GTA GTG AGA AAG GGT CGC-3′ Reverse: 5′-TGG GGC AAA TTG TTT AAG GTC TT-3′	[52]
*CHOP*	Forward: 5′-TTG CCT TTC TCC TTC GGG AC-3′ Reverse: 5′-CAG TCA GCC AAG CCA GAG AA-3′	[53]
*SOD*	Forward: 5′-GGT GTG GCC GAT GTG TCT AT-3′ Reverse:: 5′-CCT TTG CCC AAG TCA TCT GC-3′	[54]
*CAT*	Forward: 5′-TGT TGC TGG AGA ATC GGG TTC-3′ Reverse: 5′-TCC CAG TTA CCA TCT TCT GTG TA-3′	[54]
*INOS*	Forward: 5′-TGA ACT ACG TCC TGT CCC CT-3′ Reverse: 5′-CTC TTC TCT TGG GTC TCC GC-3′	[55]
*GAPDH*	Forward: 5′-TCA ACA GCG ACA CCC AC-3′ Reverse: 5′-GGG TCT CTC TCT TCC TCT TGT G-3′	[56]

## Data Availability

Data are contained within the article and Supplementary Materials.

## Supplementary Materials

The following supporting information can be downloaded at https://www.mdpi.com/article/10.3390/ph17101402/s1, Figure S1. The volcano diagram of differentially expressed genes between THP-1 and SP1-induced THP-1 cells shows 30 upregulated and 28 downregulated genes; Figure S2. The volcano diagram of differentially expressed genes between SP1-induced THP-1 (TS) and SP1-induced THP-1 treated with luteolin (TSL) shows 2012 upregulated and 560 downregulated genes; Figure S3. Original blots of luteolin inhibited the ER marker (p-PERK, CAMK2A, CHOP) in Spike-S1- induced THP-1 cells; Figure S4. Amplification curves of the ER marker (*CAMK2A* (A), *CHOP* (B), *SOD* (C), *CAT* (D), and *INOS* (E)) using RT-qPCR; Figure S5. Amplification curves of the pro-inflammatory cytokines (IL-6 (A), IL-8 (B), IL-1β (C)) using RT-qPCR; Figure S6. Original blots of luteolin inactivated the ERK/JNK/p38 signaling pathway in spike-S1-induced THP-1 cells; Table S1. The cycle threshold point (Ct) of ER marker genes were determined during the exponential phase of the PCR cycle (Amplification curves); Table S2. The cycle threshold point (Ct) of pro-inflammatory cytokines (IL-6, IL-8, IL-1β genes) were determined during the exponential phase of the PCR cycle (Amplification curves).
