# Supplementary material for: Inhibitory Effect of Luteolin on Spike S1 Glycoprotein-Induced Inflammation in THP-1 Cells via the ER Stress-Inducing Calcium/CHOP/MAPK Pathway
Source: Pharmaceuticals (Basel). 2024 Oct 20;17(10):1402. doi: 10.3390/ph17101402 (PMC11509993; doi:10.3390/ph17101402)
Supplement: Supplementary file 1 [file pharmaceuticals-17-01402-s001.zip › pharmaceuticals-3202828-supplementary.pdf]

Supplement data

Table S1. The cycle threshold point (Ct) of ER marker genes were determined during the exponential phase of the PCR cycle (Amplification curves).

Genes	Samples	Ct
CAMK2A	Non SP	30.997
	Spike S1	28.157
	2	28.615
	4.5	29.045
	9	30.146
	18	32.828
CHOP	Non SP	23.195
	Spike S1	22.107
	2	22.374
	4.5	22.401
	9	22.680
	18	24.109
SOD	Non SP	19.424
	Spike S1	18.553
	2	18.685
	4.5	18.722
	9	19.161
	18	20.320
CAT	Non SP	23.865
	Spike S1	22.658
	2	23.569
	4.5	23.085
	9	23.176
	18	24.614
INOS	Non SP	33.468
	Spike S1	31.669
	2	32.105
	4.5	32.814
	9	33.192
	18	34.341

Table S2. The cycle threshold point (Ct) of pro-inflammatory cytokines (IL-6, IL-8, IL-1β genes) were determined during the exponential phase of the PCR cycle (Amplification curves).

Genes	Samples	Ct
IL-6	Non SP	23.884
	Spike S1	21.873
	2	22.260
	4.5	23.275
	9	23.307
	18	24.256
IL-8	Non SP	17.999
	Spike S1	16.360
	2	16.870
	4.5	16.696
	9	17.133
	18	18.069
IL-1β	Non SP	29.635
	Spike S1	26.702
	2	27.567
	4.5	28.083
	9	28.835
	18	30.128

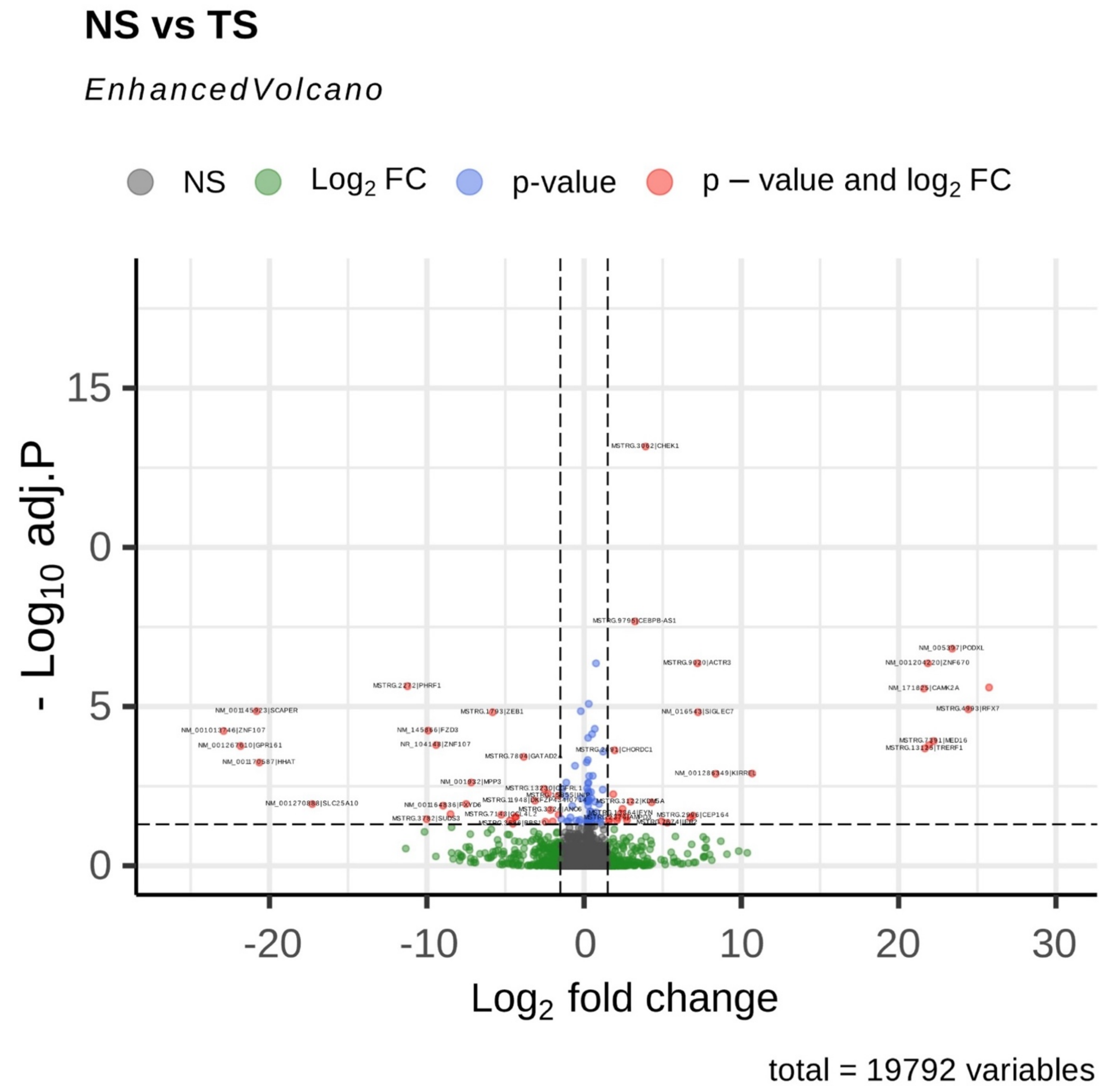

Figure S1. The volcano diagram of differentially expressed genes between THP-1 and SP1-induced THP-1 cells shows 30 upregulated and 28 downregulated genes.

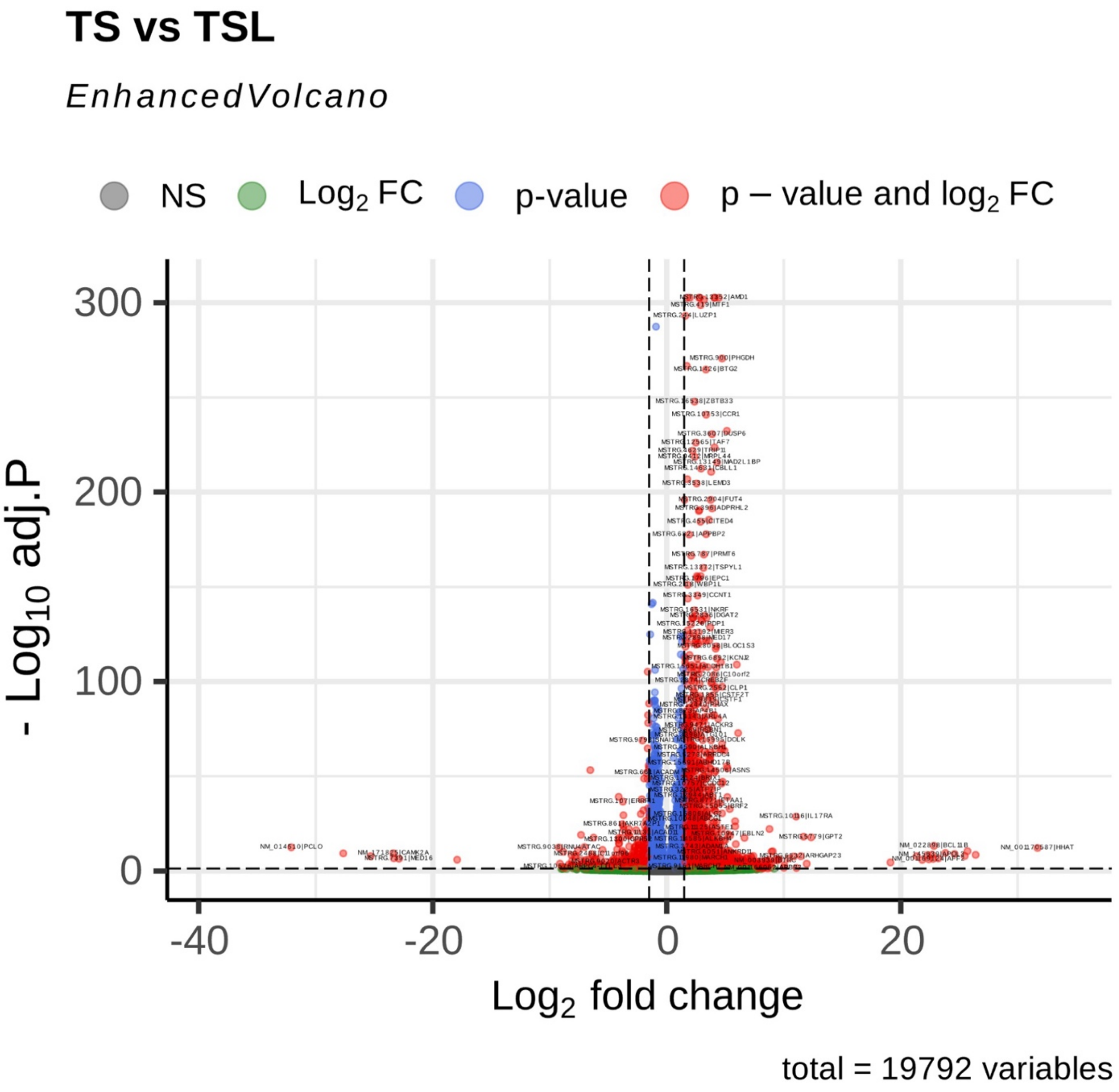

Figure S2. The volcano diagram of differentially expressed genes between SP1-induced THP-1 (TS) and SP1-induced THP-1 treated with luteolin (TSL) shows 2,012 upregulated and 560 downregulated genes.

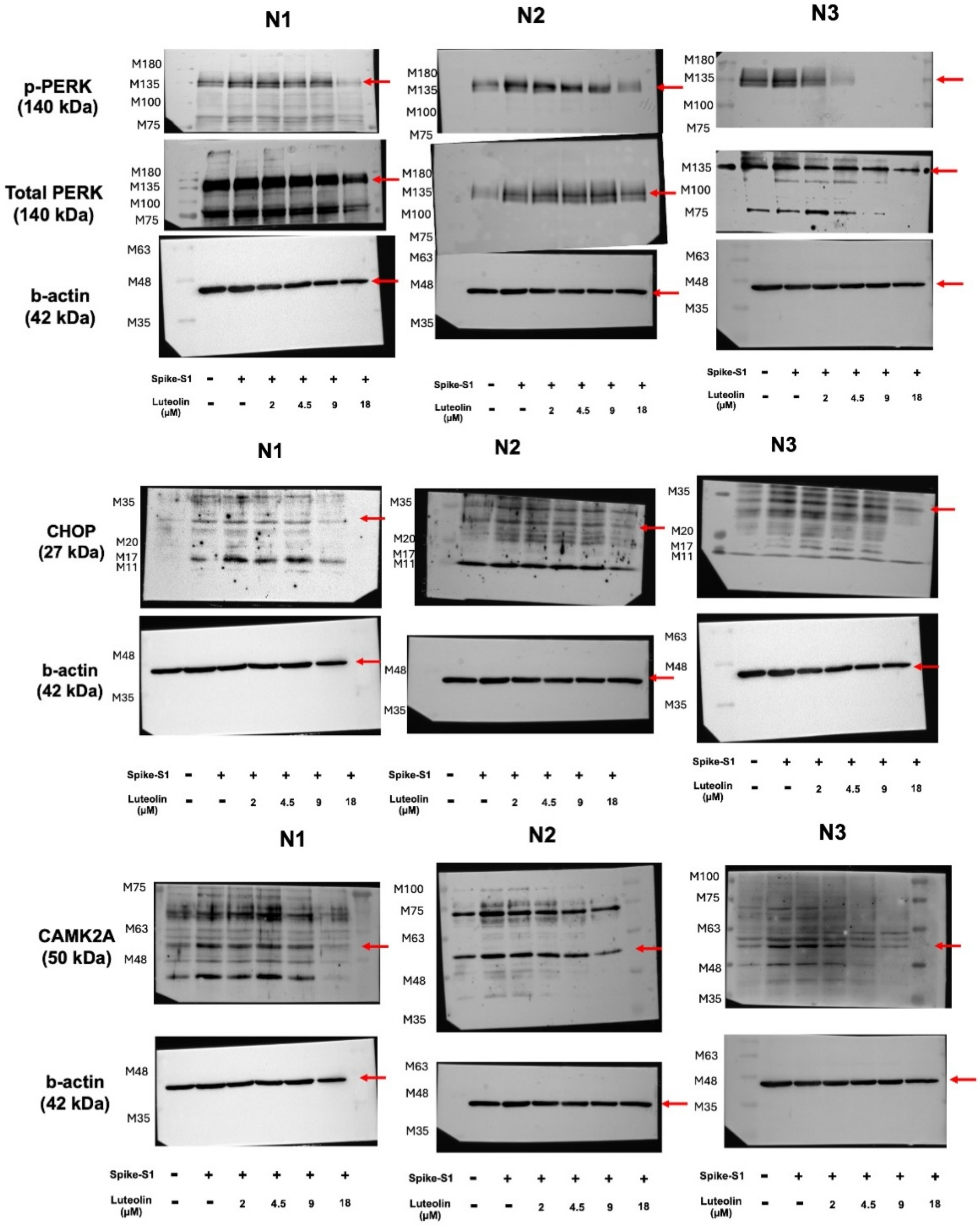

Figure S3. Original blots of luteolin inhibited the ER marker (p-PERK, CAMK2A, CHOP) in Spike-S1- induced THP-1 cells.

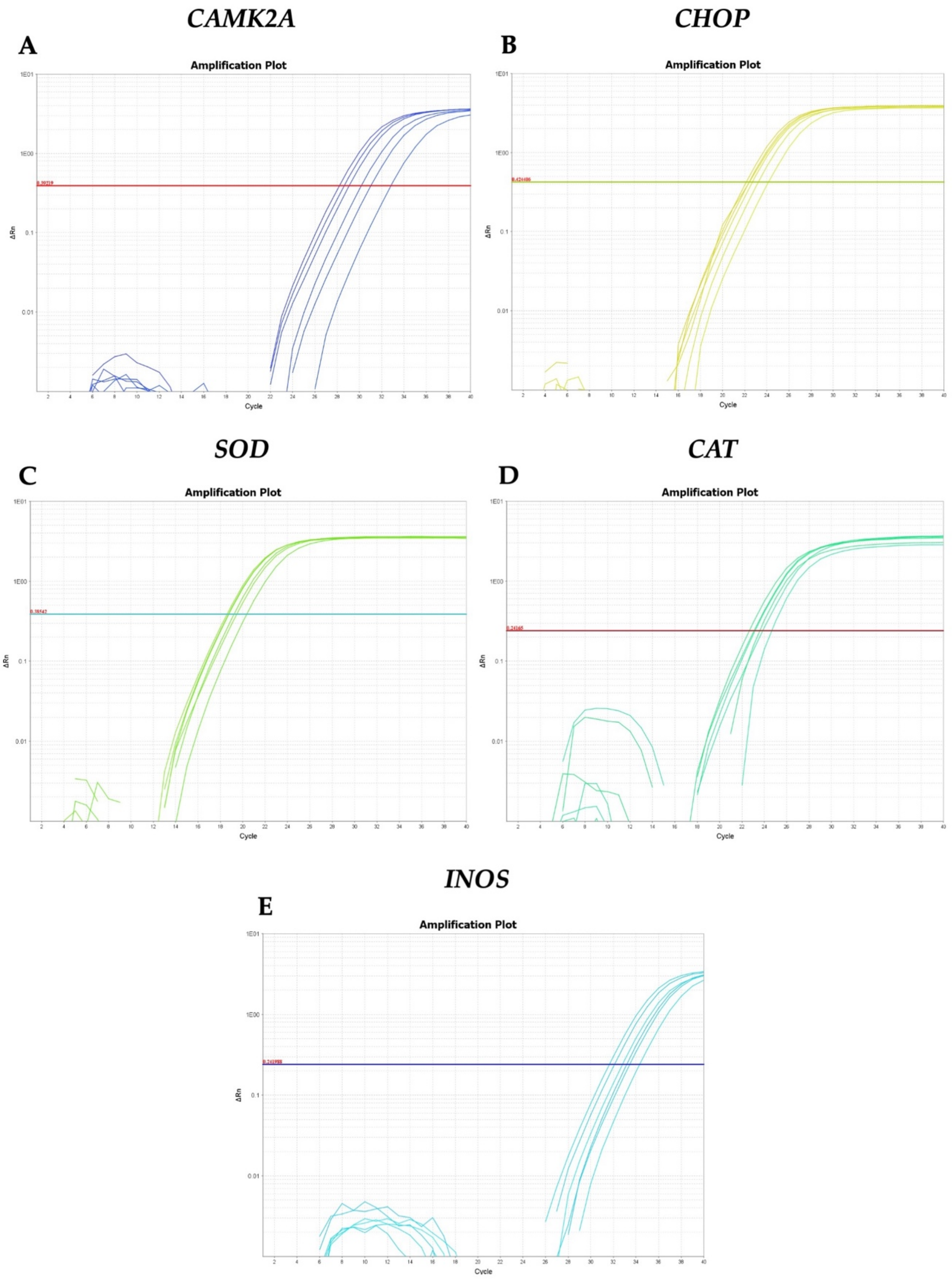

Figure S4. Amplification curves of the ER marker (CAMK2A (A), CHOP (B), SOD (C), CAT (D), and INOS (E)) using RT-qPCR

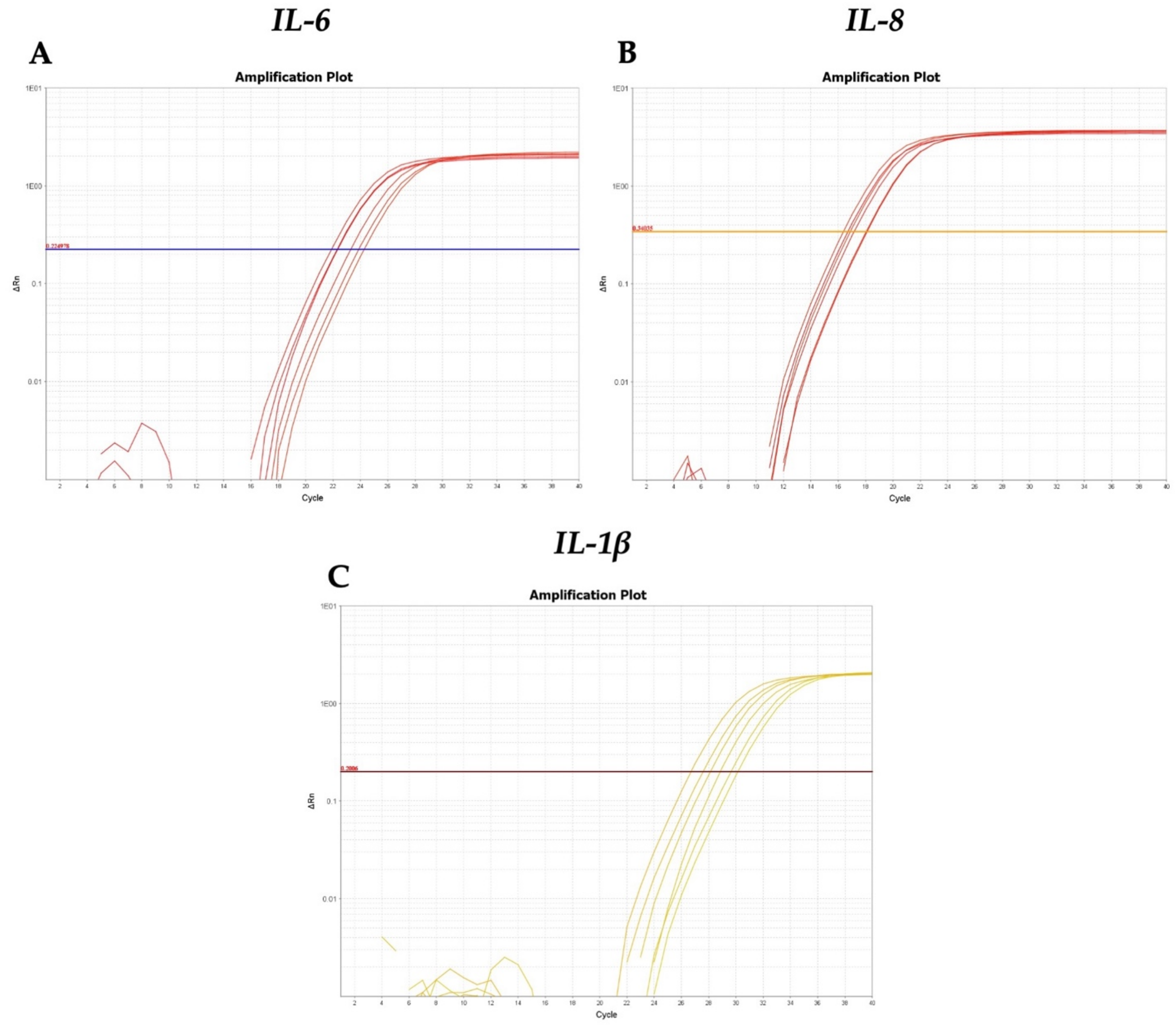

Figure S5. Amplification curves of the pro-inflammatory cytokines (IL-6 (A), IL-8 (B), IL-1β (C)) using RT-qPCR

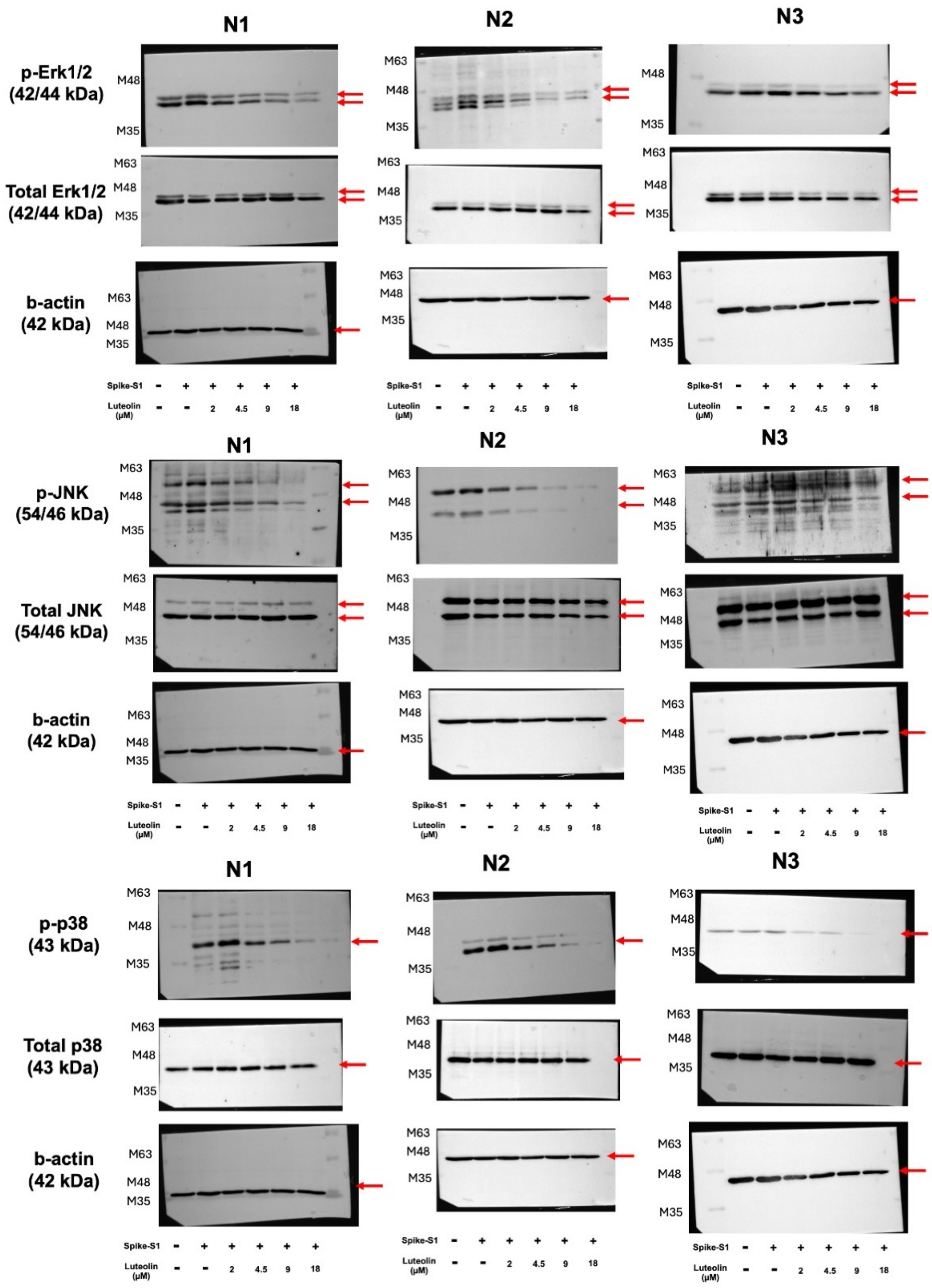

Figure S6. Original blots of luteolin inactivated the ERK/JNK/p38 signaling pathway in spike-S1- induced THP-1 cells.
